# Radiomic Signature of the Substantia Nigra on Neuromelanin‐Sensitive MRI Distinguishes Bipolar II Disorder From Unipolar Depression

**DOI:** 10.1002/brb3.71571

**Published:** 2026-07-08

**Authors:** Xinping Kuai, Dandan Shao, Shengyu Wang, Fangsong Zhang, Suzhen Zhang, Xuexue Wang, Jinhong Wang

**Affiliations:** ^1^ Department of Radiology, Shanghai Mental Health Center Shanghai Jiao Tong University School of Medicine Shanghai China; ^2^ Department of Radiology, Ruijin Hospital Shanghai Jiao Tong University School of Medicine Shanghai China; ^3^ Division of Psychotic Disorders, Shanghai Mental Health Center Shanghai Jiao Tong University School of Medicine Shanghai China

**Keywords:** bipolar disorder, neuromelanin‐sensitive MRI, radiomics, substantia nigra, support vector machine, unipolar depression

## Abstract

**Purpose:**

Early differentiation between bipolar disorder type II (BD‐II) and unipolar depression (UD) is critical yet challenging owing to overlapping depressive symptoms. This study aimed to develop and validate a radiomic signature based on substantia nigra (SN) neuromelanin‐sensitive magnetic resonance imaging (NM‐MRI) for distinguishing BD‐II, UD, and healthy controls (HCs).

**Materials and Methods:**

This secondary analysis enrolled 46 drug‐naïve BD‐II patients, 38 drug‐naïve UD patients, and 42 HCs. A total of 2854 radiomic features were extracted from manually segmented SN regions. A multi‐stage feature selection pipeline—including inter‐observer reproducibility (ICC > 0.75), false discovery rate‐corrected ANOVA, correlation analysis (|*r*| < 0.8), and least absolute shrinkage and selection operator (LASSO) regression—was used to identify a robust signature. Diagnostic performance of linear support vector machine (SVM) and multinomial logistic regression (LR) was evaluated via leave‐one‐out cross‐validation (LOOCV).

**Results:**

The multi‐stage selection yielded a parsimonious 7‐feature radiomic signature from 2854 initial candidates. For three‐class discrimination (BD‐II vs. UD vs. HC), SVM outperformed multinomial LR with higher overall accuracy (69.0% vs. 62.7%) and superior discriminative power. SVM's macro‐average AUC was 0.849 (95% CI: 0.797–0.884) and its micro‐average AUC was 0.840 (95% CI: 0.797–0.884), compared to LR's macro‐average AUC 0.812 (95% CI: 0.770–0.861) and micro‐average AUC 0.815 (95% CI: 0.770–0.861). Notably, SVM showed marked advantage in the clinically critical BD‐II versus UD classification: AUC 0.812 (95% CI: 0.720–0.905) versus LR's 0.756 (95% CI: 0.625–0.860), along with higher specificity (80.6% vs. 67.7%). The SVM model also effectively distinguished patients from HCs (UD vs. HC: AUC 0.869, 95% CI: 0.784–0.954; BD‐II vs. HC: AUC 0.860, 95% CI: 0.781–0.939).

**Conclusion:**

This radiomic signature preliminarily demonstrates potential in distinguishing BD‐II, UD, and HCs via radiomic analysis of SN NM‐MRI. The SVM model, based on the compact radiomic signature, holds promise as an objective tool for addressing BD‐II/UD diagnostic challenges, potentially supporting early intervention.

## Graphical abstract

This radiomics pipeline employed neuromelanin‐sensitive MRI to extract 2,854 features from bilateral nigral ROIs in BD‐II, UD, and HC. After Z‐score normalization, ICC (≥0.75) retention, ANOVA‐FDR (*P*<0.05) filtering, and collinearity removal (excluding feature pairs with |r| >0.8), LASSO regression selected 7 optimal features. Multinomial Logistic Regression and Linear SVM classifiers were constructed and validated via LOOCV, with confusion matrices compared to evaluate performance. This radiomic signature preliminarily demonstrates potential in distinguishing BD‐II, UD, and HCs via radiomic analysis of SN NM‐MRI. The SVM model based on the compact radiomic signature holds promise as an objective tool for addressing BD‐II/UD diagnostic challenges, potentially supporting early intervention.

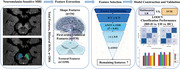

## 1 Introduction

Mood disorders, chiefly comprising unipolar depression (UD) and bipolar disorder (BD‐II), are highly prevalent, recurrent conditions that carry a substantial risk of suicide (Le et al. 2025; Sekhon and Gupta 2025; Kong et al. 2024). Affecting an estimated one in four individuals in their lifetime, they frequently lead to substantial and long‐term disability (Le‐Niculescu et al. 2021). Within this group, distinguishing BD‐II from UD poses one of psychiatry's most persistent and critical diagnostic challenges, owing to their significant symptomatic overlap. This overlap results in alarmingly high misdiagnosis rates, with approximately 40%–69% of BD‐II patients initially diagnosed with UD (Zhong et al. 2025; Hirschfeld et al. 2003). Such diagnostic errors have severe clinical ramifications: administering antidepressant monotherapy—the standard of care for UD—to BD‐II patients can induce manic switches, accelerate mood cycling, worsen the long‐term disease course, and elevate suicide risk (Phillips and Kupfer 2013; Siegel‐Ramsay et al. 2022; Jiang et al. 2024). Consequently, developing objective, neurobiologically‐based tools for early and accurate differentiation between these disorders represents a paramount and unmet need in modern psychiatry.

The substantia nigra (SN), a midbrain structure critical to the dopaminergic system, has emerged as a promising neuroanatomical target for investigating mood disorder pathophysiology (Gantz et al. 2018; Grace 2016; Ashok et al. 2017; Northoff et al. 2021). Converging evidence from neuroimaging, post‐mortem, and pharmacological studies suggests a dysregulation of the dopaminergic system in both BD and UD, albeit potentially through distinct mechanisms (Ashok et al. 2017; Belujon and Grace 2017). Neuromelanin‐sensitive magnetic resonance imaging (NM‐MRI) has emerged as a novel and powerful technique that provides an in vivo proxy for the integrity of dopaminergic and noradrenergic neurons, as neuromelanin is a byproduct of catecholamine synthesis and accumulates in these neurons over a lifetime (Trujillo et al. 2024). Our prior conventional region of interest (ROI) analysis using NM‐MRI successfully demonstrated group‐level SN alterations in mood disorders (Kuai et al. 2024), laying the groundwork for the current investigation. However, conventional ROI analyses, which often rely on summary measures like mean signal intensity, are limited in their sensitivity to the subtle, spatially heterogeneous microstructural changes that may underpin the distinct neuropathologies of BD‐II and UD.

Radiomics, a high‐throughput data‐driven approach, offers a powerful solution to this limitation (Lambin et al. 2017; Lambin et al. 2012). It involves the automated extraction of a vast array of sub‐visual, quantitative features from medical images, transforming them into mineable, high‐dimensional data (Gillies et al. 2016). These features, which encode information about textures, statistical measures, shape, and intensity features, can quantify intra‐regional tissue patterns that are imperceptible to the human eye (Shinde et al. 2019). This approach holds significant promise for enhancing the diagnosis and prognosis of psychiatric disorders (Alizadeh et al. 2023). For instance, a novel radiomics‐based feature, the high‐level morphometric similarity network derived from T1‐weighted MRI, has been shown to improve the classification of BD and UD when combined with conventional GMV features (Sun et al. 2025). Current radiomics research in psychiatry relies primarily on T1‐weighted imaging and functional MRI, while the clinical utility of other sequences (e.g., T2‐weighted, FLAIR, and post‐contrast T1‐weighted imaging) remains unelucidated (Alizadeh et al. 2023). Exploring these underutilized sequences could potentially improve the technical performance of radiomic models. Notably, NM‐MRI—a sequence directly linked to dopamine system dysfunction—has yet to undergo comprehensive radiomic analysis for distinguishing BD‐II from UD.

Building on our prior findings, we hypothesized that a radiomic signature derived from NM‐MRI of the SN would capture distinct multivariate patterns of microstructural heterogeneity, enabling accurate differentiation among BD‐II, UD, and healthy controls (HCs). Furthermore, we hypothesized that the linear support vector machine (SVM) model would outperform multinomial logistic regression (LR) in this high‐dimensional, small‐sample setting, particularly in the clinically critical differentiation between BD‐II and UD. To test these hypotheses, we employed a rigorous multi‐stage feature selection pipeline to distill a robust and non‐redundant set of features from an initial high‐dimensional dataset for diagnostic model construction and validation.

## 2 Materials and Methods

### 2.1 Participants

This study is a secondary analysis of data from our prior published work (Kuai et al. 2024), which comprehensively detailed the original participant recruitment, screening criteria, and assessment protocols. Briefly, the study included 46 patients with BD‐II, 38 patients with UD, and 42 HCs, with all groups matched for age and gender. Eligible patients were drug‐naïve individuals experiencing their first depressive episode. For BD‐II patients, this first depressive episode occurred after an initial hypomanic episode. Diagnoses were established according to the Diagnostic and Statistical Manual of Mental Disorders, Fourth Edition (DSM‐IV) criteria using the Structured Clinical Interview for DSM Disorders (SCID). At MRI acquisition, all UD patients were in a current major depressive episode with no lifetime history of mania or hypomania, whereas all BD‑II patients were in a current major depressive episode and free of mania, hypomania, or mixed symptoms. Clinical severity, assessed by the 24‐item Hamilton Depression Rating Scale (HDRS), exceeded a score of 20 for all patients. HCs were required to have no personal or family history of psychiatric illness. Key exclusion criteria included comorbid neurological or physical conditions, contraindications for MRI, and prior treatment. The study was conducted with the approval of the Shanghai Mental Health Center Ethics Committee, and all participants provided written informed consent.

### 2.2 Neuroimaging Data Acquisition and Data Preprocessing

The neuromelanin‐sensitive MRI acquisition parameters were identical to those reported previously (Kuai et al. 2024). In summary, imaging was performed on a 3 T MAGNETOM Verio scanner (Siemens Healthcare, Erlangen, Germany) with a 32‐channel head coil, using a 2D‐GRE sequence with magnetization transfer contrast (MTC). In the current radiomics analysis, we employed the predefined SN ROIs established in our previous study (Kuai et al. 2024). These ROIs, which had been manually segmented by two experienced radiologists using MRIcroGL software, were identical to those utilized in the prior conventional analysis. Initial attempts to define SN ROIs using a group‐averaged template resulted in suboptimal alignment and segmentation. We therefore performed individualized manual segmentation to ensure precise anatomical delineation. To mitigate intensity inhomogeneity and standardize intensity scales, all MRI scans underwent N4 bias field correction and image intensity normalization. Detailed acquisition and preprocessing parameters are provided in the Supporting Information.

### 2.3 Radiomics Feature Extraction

A comprehensive radiomic analysis was conducted utilizing the Radiomics Intelligent Analysis Toolkit (RIAS) (Li et al. 2020). Feature extraction was performed for each subject resulting in a total of 2854 features derived from bilateral ROIs. The pipeline comprised 17 shape features calculated from the original segmentation. First‐order statistical features (*n* = 19 per image type) and textural features—including those from gray level co‐occurrence matrix (GLCM, *n* = 24), gray level dependence matrix (GLDM, *n* = 14), gray level run length matrix (GLRLM, *n* = 16), gray level size zone matrix (GLSZM, *n* = 16), and neighboring gray tone difference matrix (NGTDM, *n* = 5)—were computed across 15 image types. These types encompassed the original image, filtered images (exponential, logarithmic, square, square root, Laplacian of Gaussian with *σ* = 1.0 and 3.0 mm), and eight wavelet‐decomposed images. Per ROI, this methodology produced 285 first‐order features and 360, 210, 240, 240, and 75 features for the GLCM, GLDM, GLRLM, GLSZM, and NGTDM classes, respectively.

### 2.4 Feature Selection

A multi‐stage feature selection pipeline was implemented to identify a robust and discriminative radiomic signature. First, all features were standardized using *Z*‐score normalization prior to reproducibility assessment. Inter‐observer reproducibility was assessed based on two independent manual segmentations of the SN. Only features with intraclass correlation coefficient (ICC) ≥ 0.75 were kept to ensure stability against ROI drawing variability. Subsequently, analysis of variance (ANOVA) combined with false discovery rate (FDR) correction (*p* < 0.05) was used to select features showing significant associations with the groups. To mitigate multicollinearity, highly correlated features (absolute Pearson's correlation coefficient, *r* > 0.8) were eliminated, giving priority to those with greater ANOVA significance. Finally, the least absolute shrinkage and selection operator (LASSO) regression was applied to the resulting non‐redundant set. The optimal regularization parameter (*λ*) was determined via 10‐fold cross‐validation, selecting the value that minimized multinomial deviance. This process selected the most parsimonious and predictive feature combination for the final model.

### 2.5 Diagnostic Model Construction and Validation

Two machine learning algorithms were implemented: a linear SVM and a multinomial LR model. Hyperparameter optimization was performed using leave‐one‐out cross‐validation (LOOCV), involving the regularization parameter C (ranging from 0.001 to 100) for SVM and the decay parameter (values of 0, 0.1, and 0.5) for LR model. A fully nested LOOCV framework was also implemented (see Supporting Information).

### 2.6 Statistical Analysis

Model performance was assessed using accuracy, sensitivity, specificity, positive predictive value (PPV), negative predictive value (NPV), and the F1 score for each diagnostic category (BD‐II, UD, and HCs). The F1 score ranges from 0 to 1. It integrates sensitivity and PPV for a comprehensive evaluation, and a higher score represents better performance. The area under the receiver operating characteristic curve (AUC) was computed for one‐versus‐rest, one‐versus‐one, and micro‐average analyses. The 95% confidence intervals (CIs) for AUC were calculated using the DeLong method based on LOOCV predicted probabilities. All analyses were conducted in R version 4.5.1 with a fixed random seed to ensure reproducibility. The core R packages used were as follows: caret, glmnet, e1071, and nnet for model development; pROC and MLmetrics for performance evaluation; irr for inter‐rater reliability analysis; and tidyverse for data manipulation and preprocessing.

## 3 Results

### 3.1 Demographic and Clinical Characteristics

As reported previously (Kuai et al. 2024), no significant differences were observed in age (BD‐II: 26.67 ± 5.40; UD: 27.79 ± 6.93; HC: 28.26 ± 5.57; *p* = 0.44), gender distribution (*p* = 0.10), or years of education (*p* = 0.23) among the groups. HDRS‐24 scores did not differ significantly between BD‐II and UD patients (28.98 ± 8.73 vs. 31.24 ± 12.20; *p* = 0.33).

### 3.2 Feature Selection

**FIGURE 1:**
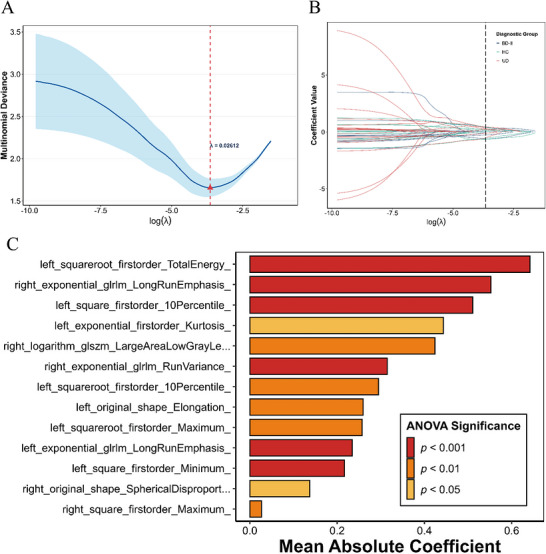
(A) LASSO cross‐validation curve: Results of 10‐fold cross‐validation showing the relationship between multinomial deviance and log(lambda). The optimal lambda (indicated by the red dashed line) corresponds to the value that minimizes deviance, achieving a balance between model complexity and predictive accuracy. (B) Coefficient trajectories of all features across a range of regularization parameters. Colored lines represent different diagnostic groups (BD‐II: blue, UD: red, HC: green). Features retained in the final model are those with non‐zero coefficients at the optimal lambda (marked by the black dashed line). (C) Feature importance ranking: Features are ordered by their mean absolute coefficient values across all classes. Color shading reflects statistical significance based on ANOVA results.

**FIGURE 2:**
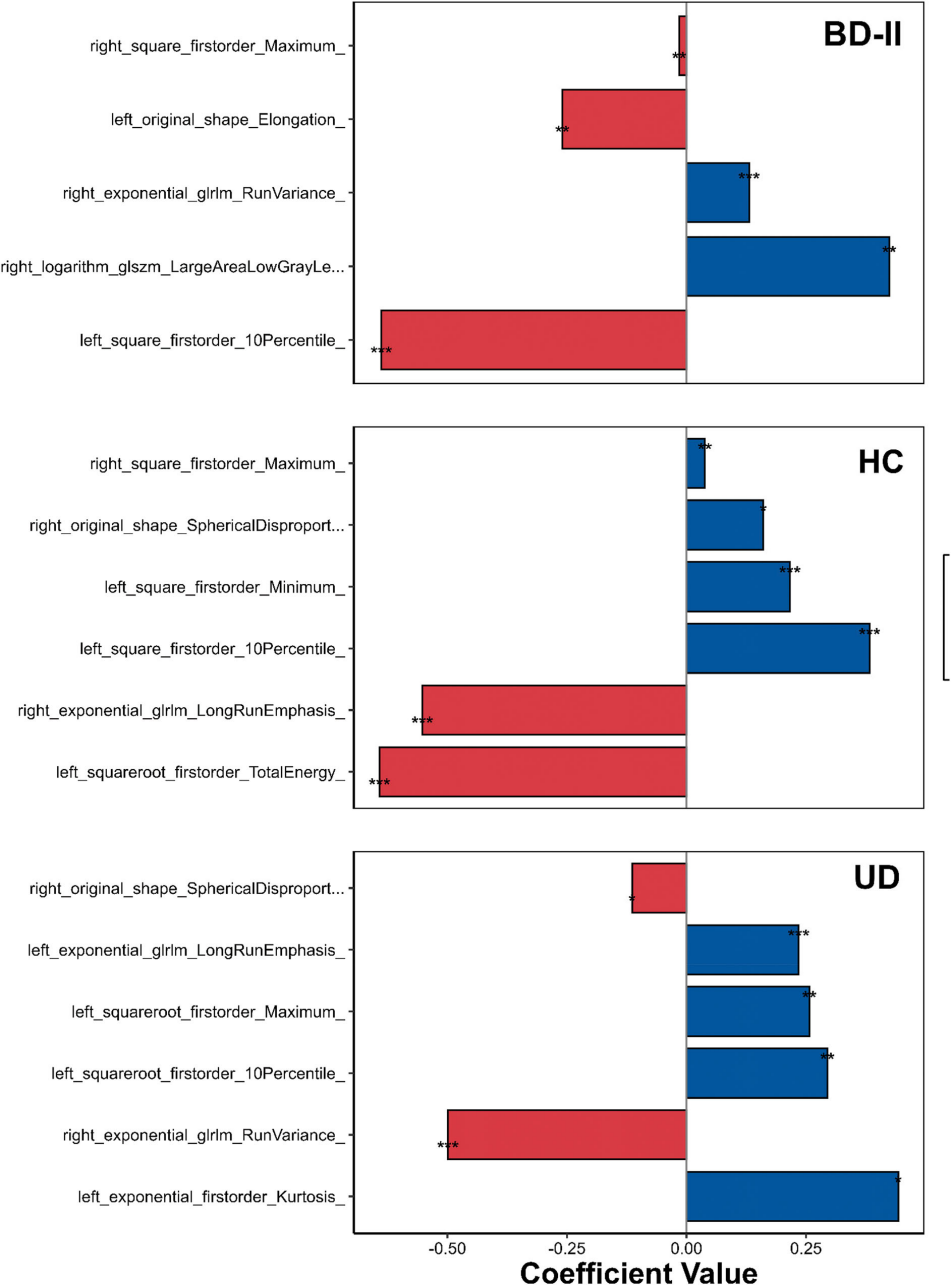
Class‐specific LASSO coefficients. This panel illustrates the final regularized coefficients for features selected by the LASSO regression across the three diagnostic classes. The direction (positive: blue; negative: red) and magnitude of the coefficients reflect each feature's contribution to predicting the respective diagnostic category. Statistical significance from prior ANOVA analysis is denoted by asterisks (*p < 0.05, **p < 0.01, ***p < 0.001).

**FIGURE 3:**
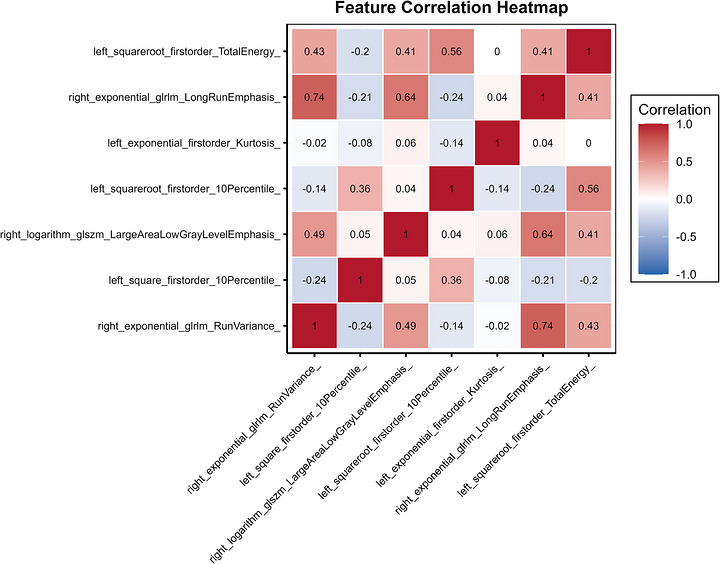
Feature correlation heatmap: This heatmap depicts inter‐feature relationships, where red denotes positive correlations and blue indicates negative correlations. The predominance of light hues demonstrates that the majority of correlations are weak to moderate (absolute value < 0.8), suggesting low multicollinearity among the selected feature set.

Based on a multi‐stage feature selection pipeline, a robust radiomic signature was successfully constructed. The process commenced with an evaluation of inter‐observer reproducibility, which identified 1019 features with good consistency (ICC > 0.75) from an initial set of 2854. Subsequent ANOVA analysis with FDR correction revealed 129 significant features (*p* < 0.05). Correlation‐based screening of these 129 features removed 109 highly correlated variables (|*r*| > 0.8), yielding 20 non‐redundant features. Finally, LASSO regression with 10‐fold cross‐validation was applied to the set of 20 candidate features. Using the optimal regularization parameter (*λ* = 0.026), which minimized multinomial deviance (1.657), 13 features were retained, achieving an optimal balance between model parsimony and predictive performance (Figures 1 and 2). To enhance model generalizability, given the limited sample size, we selected the seven features exhibiting the largest absolute LASSO coefficients: right_exponential_glrlm_Run Variance, left_square_first order_10Percentile, right_logarithm_glszm_Large Area Low Gray Level Emphasis, left_squareroot_first order_10Percentile, left_exponential_first order_Kurtosis, right_exponential_glrlm_Long Run Emphasis, and left_squareroot_first order_Total Energy. The correlation structure among the selected features is shown in Figure 3. The resulting correlation heatmap indicates an acceptable degree of mutual independence among the predictors, supporting the statistical robustness of the final model.

### 3.3 Model Performance and Discriminative Ability

**FIGURE 4:**
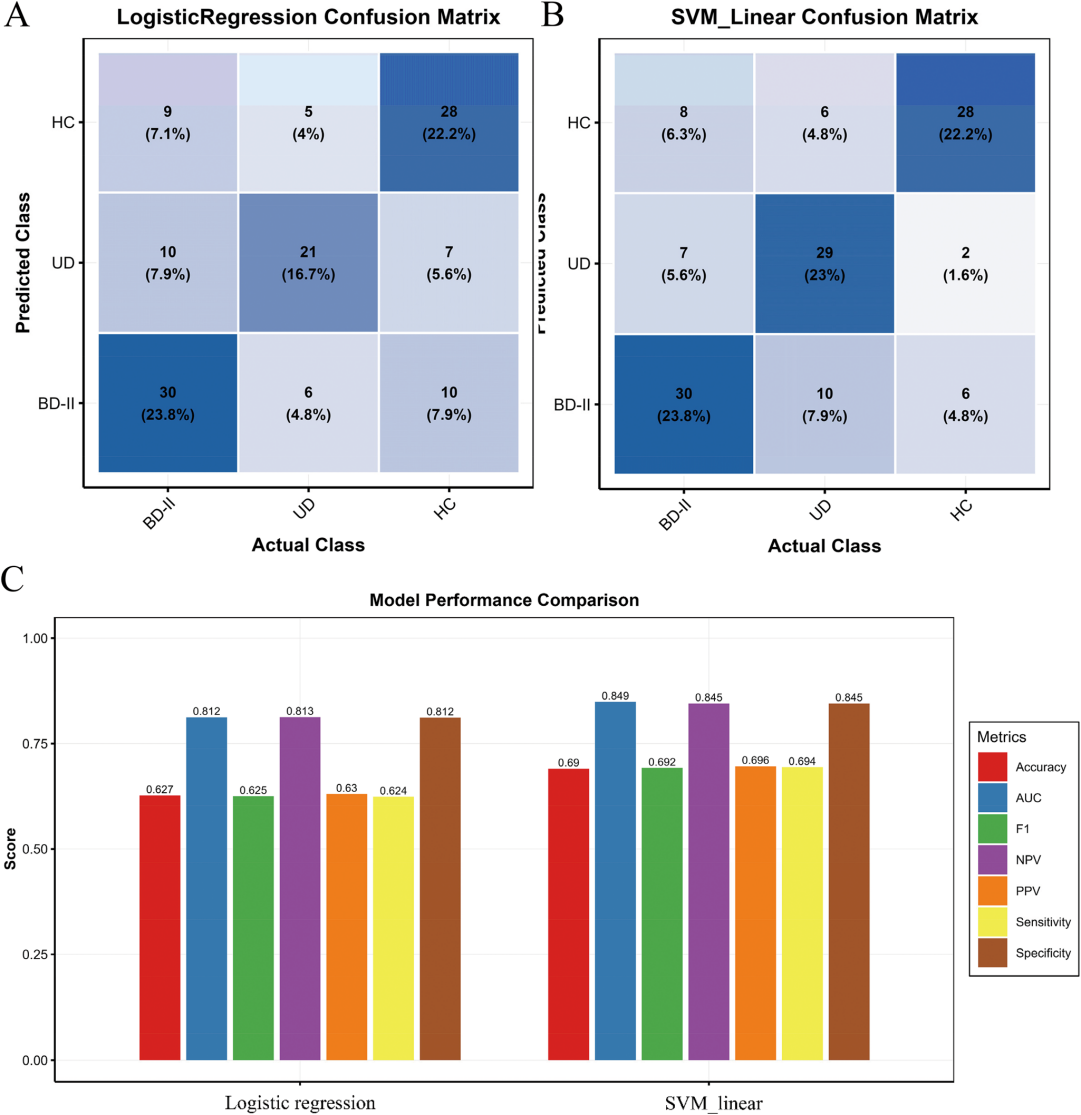
Model performance evaluation. (A, B) Confusion matrices from leave‐one‐out cross‐validation for the (A) logistic regression and (B) support vector machine (SVM) models. Values in cells show the count of samples with the corresponding percentage of the total in parentheses. The diagonal (from bottom‐left to top‐right) represents correct classifications. (C) A comparative evaluation of multiple classification models across key performance metrics—including accuracy, area under the curve (AUC), F1 score, negative predictive value (NPV), positive predictive value (PPV), sensitivity, and specificity—illustrated using grouped bar plots.

The discriminative performance of the SVM and multinomial LR classifiers was evaluated using LOOCV, with the aggregated prediction results visualized in the confusion matrices (Figure 4A,B). The SVM model demonstrated superior performance compared to the LR model. The overall results for the three‐class classification (BD‐II vs. UD vs. HC) are summarized in Table 1. SVM demonstrated superior performance over LR model in psychiatric differential diagnosis, achieving notably higher overall accuracy (69.0% vs. 62.7%) and discriminative power, as reflected in macro‐average AUC (0.849 vs. 0.812) and micro‐average AUC (0.840 vs. 0.815). This enhanced diagnostic reliability was further supported by SVM's higher F1 score (69.2% vs. 62.5%), indicating a better balance between PPV and sensitivity (Figure 4C).

**TABLE 1:** Comparative performance metrics of machine learning models for psychiatric disorder classification.

Model	Acc (%)	Sens (%)	Spec (%)	PPV (%)	NPV (%)	F1 score (%)	AUC (95% CI)
SVM	69.0	69.4	84.5	69.6	84.5	69.2	0.849 (0.797–0.884)
LR	62.7	62.4	81.2	63	81.3	62.5	0.812 (0.770–0.861)

*Note*: All values represent mean performance scores derived from leave‐one‐out cross‐validation.

Abbreviations: Acc, accuracy; AUC, area under the curve; CI, confidence interval; LR, logistic regression; NPV, negative predictive value; PPV, positive predictive value; Sens, sensitivity; Spec, specificity; SVM, support vector machine.

**FIGURE 5:**
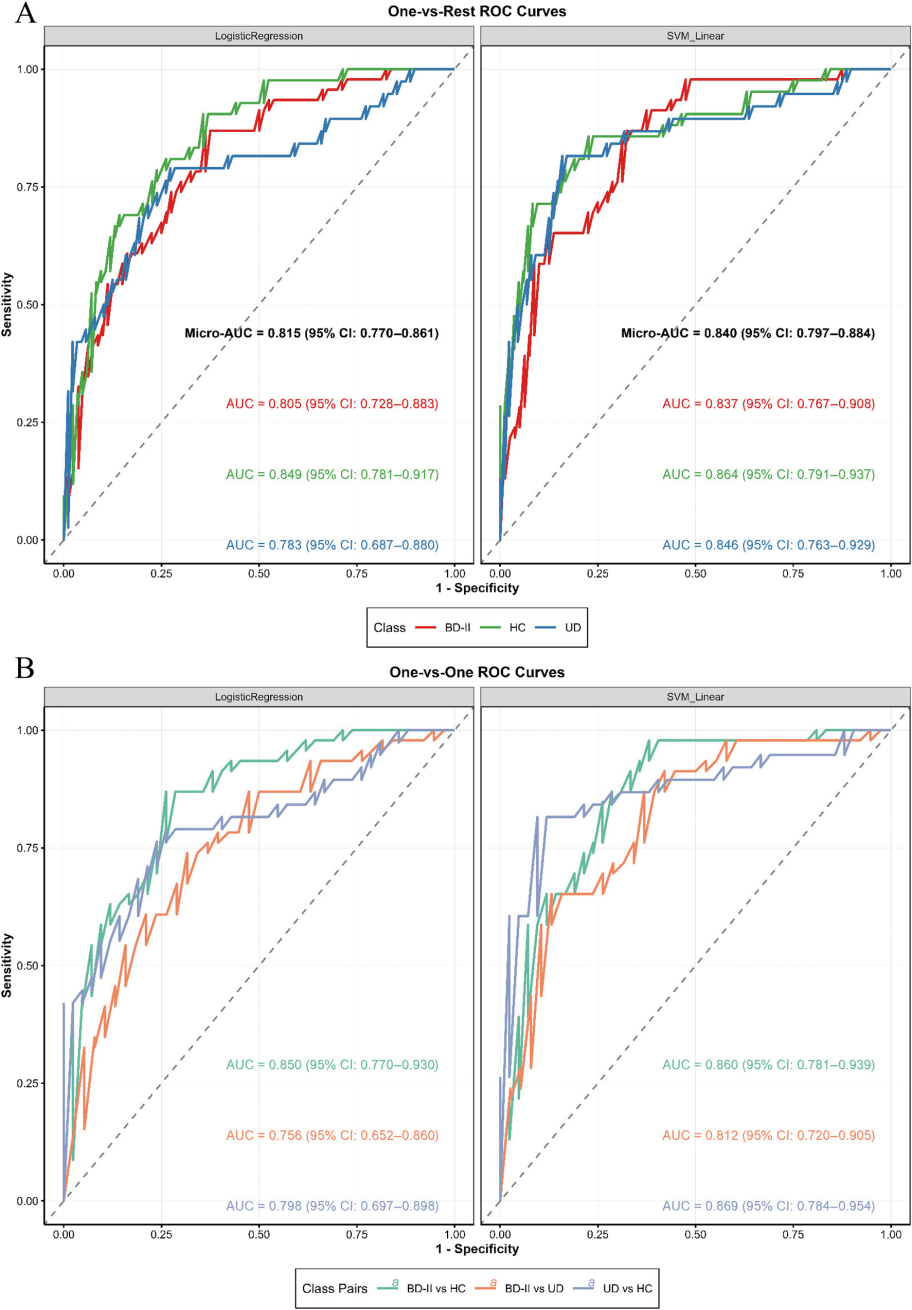
(A) One‐versus‐rest ROC analysis: Receiver operating characteristic (ROC) curves for classifying each class against all others, showing the area under the curve (AUC) with 95% confidence intervals and the micro‐average AUC. (B) One‐versus‐one ROC analysis: Pairwise ROC curves (BD‐II vs. HC, UD vs. HC, and BD‐II vs. UD) with their respective AUC values.

In the one‐versus‐rest analysis (Figure 5A), SVM achieved higher AUC values for all three diagnostic categories (BD‐II: 0.837 vs. 0.805; UD: 0.846 vs. 0.783; HC: 0.864 vs. 0.849), with Table 2 confirming its advantages in overall accuracy for HC (82.5% vs. 75.4%) and UD (80.2% vs. 77.8%) classification. The performance advantage was particularly pronounced in pairwise differentiations (Figure 5B). SVM excelled in distinguishing patient groups from HC, achieving AUCs of 0.869 (UD vs. HC) and 0.860 (BD‐II vs. HC), substantially outperforming LR model (0.798 and 0.850, respectively). Table 3 further demonstrates SVM's exceptional sensitivity (93.5% vs. 75.0%) and NPV (93.3% vs. 80.0%) for UD versus HC classification. Most notably, in the clinically challenging BD‐II versus UD differentiation, SVM maintained superior balanced performance with an AUC of 0.812 versus 0.756 for LR model. While LR model showed higher sensitivity (83.3% vs. 75.0%), SVM achieved markedly better specificity (80.6% vs. 67.7%) and overall accuracy (77.6% vs. 76.1%), representing a more reliable diagnostic profile for this critical distinction. In the more stringent nested LOOCV, SVM achieved an accuracy of 61.1% and a multiclass AUC of 0.734 (Supporting Information).

**TABLE 2:** Class‐level performance metrics for SVM and logistic regression models.

Class	Model	Acc (%)	Sens (%)	Spec (%)	PPV (%)	NPV (%)	F1 score (%)	AUC (95% CI)
BD‐II	SVM	75.4	65.2	81.3	66.7	80.2	65.9	0.837 (0.767–0.908)
	LR	72.2	65.2	76.3	61.2	79.2	63.2	0.805 (0.728–0.883)
UD	SVM	80.2	76.3	81.8	64.4	88.9	69.9	0.846 (0.763–0.929)
	LR	77.8	55.3	87.5	65.6	81.9	60.0	0.783 (0.687–0.880)
HC	SVM	82.5	66.7	90.5	77.8	84.4	71.8	0.864 (0.791–0.937)
	LR	75.4	66.7	79.8	62.2	82.7	64.4	0.849 (0.781–0.917)

Abbreviations: Acc, accuracy; AUC, area under the curve; BD‐II, bipolar disorder type II; CI, confidence interval; HC, healthy controls; LR, logistic regression; NPV, negative predictive value; PPV, positive predictive value; Sens, sensitivity; Spec, specificity; SVM, support vector machine; UD, unipolar depression.

**TABLE 3:** Pairwise classification performance between diagnostic groups.

Comparison	Model	Acc(%)	Sens(%)	Spec(%)	PPV(%)	NPV(%)	F1 score(%)	AUC(95% CI)
BD‐II vs. HC	SVM	80.6	83.3	77.8	78.9	82.4	81.1	0.860 (0.781–0.939)
	LR	75.3	75	75.7	76.9	73.7	75.9	0.850 (0.770–0.930)
UD vs. HC	SVM	87.7	93.5	82.4	82.9	93.3	87.9	0.869 (0.784–0.954)
	LR	80.3	75	84.8	80.8	80	77.8	0.798 (0.697–0.898)
BD‐II vs. UD	SVM	77.6	75	80.6	81.1	74.4	77.9	0.812 (0.720–0.905)
	LR	76.1	83.3	67.7	75	77.8	78.9	0.756 (0.652–0.860)

Abbreviations: Acc, accuracy; AUC, area under the curve; BD‐II, bipolar disorder type II; CI, confidence interval; HC, healthy controls; LR: logistic regression; NPV, negative predictive value; PPV, positive predictive value; Sens, sensitivity; Spec, specificity; SVM, Support Vector Machine; UD, unipolar depression.

## 4 Discussion

This study presents a novel radiomic approach utilizing features derived from neuromelanin‐sensitive MRI of the SN to differentiate BD‐II, UD, and HCs. A multi‐stage feature selection pipeline identified a parsimonious set of seven features, and SVM demonstrated superior performance across all evaluation frameworks. Comparative analyses revealed that SVM consistently outperformed traditional LR, achieving significantly higher overall accuracy (69.0% vs. 62.7%) and discriminative power (macro‐average AUC: 0.849 vs. 0.812). SVM also exhibited better performance in micro‐average analysis (AUC: 0.840 vs. 0.815), confirming its enhanced capability in handling class‐imbalanced data. Furthermore, SVM showed robust performance in three‐class classification and effectively discriminated patient groups from HCs (UD vs. HC: AUC = 0.869 vs. 0.798; BD‐II vs. HC: AUC = 0.860 vs. 0.850), as well as BD‐II from UD (AUC = 0.812 vs. 0.756). SVM's substantially higher specificity (80.6% vs. 67.7% for BD‐II vs. UD) and balanced performance profile suggest its potential to reduce overdiagnosis of bipolarity in depressive presentations.

A key challenge in early mood disorders is the substantial symptomatic overlap between BD‐II and UD during depressive episodes, leading to misdiagnosis (with approximately 40%–69% of BD‐II cases being incorrectly diagnosed as UD), delayed treatment, worsened progression, and elevated suicide risk (Zhong et al. 2025; Hirschfeld et al. 2003; Phillips and Kupfer 2013; Siegel‐Ramsay et al. 2022; Jiang et al. 2024). Bridging this diagnostic gap has been a central pursuit in psychiatry (Insel et al. 2010). Building on our previous finding that conventional ROI analyses can detect group‐level SN pathology in mood disorders (Kuai et al. 2024), we adopted a radiomics approach to overcome their limited sensitivity to spatial‐textural heterogeneity. This data‐driven technique decodes sub‐visual features of texture, intensity, and morphology from high‐dimensional data, thereby quantifying intra‐regional heterogeneity and providing a superior tool for characterizing SN pathology (Gillies et al. 2016; Kumar et al. 2012). The multi‐stage feature selection pipeline, incorporating reproducibility analysis, statistical significance, and collinearity checks, was crucial for distilling the initial 2854 features down to a robust and non‐redundant signature of seven features. This rigorous process mitigates the high‐dimensionality risk inherent in radiomics studies (Lambin et al. 2017).

The biological plausibility of our findings is anchored in the central role of the dopaminergic system, originating in the SN and ventral tegmental area, in the pathophysiology of mood disorders (Gantz et al. 2018; Grace 2016; Ashok et al. 2017; Northoff et al. 2021). These radiomic features are not derived from conventional neuromelanin MRI metrics such as contrast‐to‐noise ratio (CNR) or mean signal intensity. Instead, they represent multi‐level quantitative descriptors that comprehensively characterize the SN on neuromelanin‐sensitive MRI. Specifically, they include: (1) first‐order statistics that describe the overall distribution of image signal intensity; (2) texture features that reflect spatial heterogeneity, local pixel correlation, and tissue microstructure; and (3) morphological features that describe the shape and structural properties of the SN. Together, these features capture subtle, subvisual tissue differences that cannot be detected by standard CNR or visual assessment alone. The seven features span first‐order statistical and textural domains: first‐order parameters (e.g., 10th percentile, kurtosis, and total energy) quantify global signal intensity distributions linked to tissue composition, while GLRLM/GLSZM textural features (e.g., run variance, long‐run emphasis, and large‐area low‐gray level emphasis) decode local structural continuity via spatial inter‐pixel relationships (Lambin et al. 2012, Shinde et al. 2019). This multifaceted strategy enables the signature to identify disease‐specific SN alterations eluding conventional imaging. Our findings provide novel imaging evidence for distinct SN pathophysiological processes in BD‐II and UD patients, with radiomic features’ discriminative power likely reflecting microstructural differences (e.g., neuromelanin concentration, iron deposition, and cytoarchitectonic changes) undetectable by conventional MRI. In sum, our model addresses the critical BD‐II/UD diagnostic gap and generates testable hypotheses regarding the two disorders’ distinct neurobiology.

The observed performance gap, where linear SVM consistently surpasses multinomial LR, has a solid theoretical basis in high‐dimensional, small‐sample settings (Huang et al. 2018; Xu et al. 2019; Furey et al. 2000)—the challenging “small *n*, large *p*” domain where SVMs naturally excel (Huynh et al. 2020). This difference originates in their learning principles. LR, as a parametric model, uses maximum likelihood estimation. This method becomes unstable with high feature‐to‐sample ratios, leading to high variance and overfitting (Babyak 2004). SVM, based on structural risk minimization, bypasses full distribution modeling to find the maximum‐margin separating hyperplane (Chapelle et al. 1999). This margin‐based approach is fundamental to its robust generalization, as it inherently limits model complexity and mitigates the impact of noise in high dimensions (Statnikov et al. 2008).

Despite these promising results and methodological strengths, several limitations warrant consideration. First, our study was limited by a relatively small, single‐center, drug‐naïve, and relatively young sample size, which is typical in initial neuroimaging studies of well‐characterized patients. Accordingly, the present findings may not generalize to medicated patients or real‐world clinical settings. Notably, the SVM classifier demonstrated superior performance, highlighting its suitability for high‐dimensional data with limited samples, where its maximum‐margin principle mitigates overfitting and improves generalization. Our adoption of LOOCV further reinforced the internal validity of the radiomic findings. Despite these strengths, future work with larger, multi‐center cohorts is crucial to verify the model's generalizability and investigate potential subgroup effects. Second, the exclusive focus on the SN, while well‐justified, limits the coverage of other brain regions implicated in mood disorders. Although the locus coeruleus (LC) was visible on our neuromelanin‐sensitive MRI scans, this nucleus has an inherently small anatomical volume and presented poor visualization and ambiguous boundaries across participants. To guarantee the reliability of radiomic quantification, we did not include the LC in the current analysis. Future studies should adopt high‐resolution imaging protocols specifically optimized for the LC. In addition, integrating multi‐region radiomic signatures or combining structural radiomics with functional connectivity data would further improve diagnostic performance. Finally, while radiomics provides powerful mathematical descriptors, the precise neurobiological correlations of the selected features are not yet fully understood. The selected radiomic features may reflect alterations in neuromelanin content, iron deposition, or tissue cytoarchitecture. However, these interpretations remain speculative and are presented as working hypotheses in this study. Future studies integrating multi‐modal imaging, such as quantitative susceptibility mapping, could help elucidate the biological underpinnings of the radiomic signature.

## 5 Conclusions

This work provides evidence that radiomic analysis of neuromelanin‐sensitive MRI can distinguish BD‐II, UD, and HCs with high accuracy, particularly when modeled using SVM. These findings highlight the utility of data‐driven neuroimaging approaches in capturing subtle brain phenotypes and offer a foundation for developing objective biomarkers to improve diagnostic accuracy in mood disorders. Future research should prioritize external validation, longitudinal follow‐up, and integration with multi‐modal data to realize the full potential of radiomics in clinical practice.

## Author Contributions

**Xinping Kuai**: methodology, conceptualization, data curation, software, writing – original draft, writing – review and editing. **Dandan Shao**: data curation, investigation, software, writing – original draft. **Shengyu Wang**: data curation, investigation, software, writing – original draft. **Fangsong Zhang**: data curation. **Suzhen Zhang**: funding acquisition, software, data curation. **Xuexue Wang**: supervision, data curation. **Jinhong Wang**: supervision, data curation.

## Funding

This work was supported by the Multidisciplinary Cross Research Foundation of Shanghai Jiao Tong University (YG2024QNA55).

## Ethics Statement

The authors declare that the work described has been carried out in accordance with the Declaration of Helsinki of the World Medical Association, revised in 2013, for experiments involving humans.

## Conflicts of Interest

The authors declare no conflicts of interest.

## Supporting information




**Supplementary Materials**: brb371571‐sup‐0001‐SuppMat.docx

## Data Availability

The data that support the findings of this study are available from the corresponding author upon reasonable request. The code used in this work is standard routine code and can also be obtained from the corresponding author on request.

## References

[brb371571-bib-0001] Alizadeh, M. , M. Tanwar , A. H. Sarrami , R. Shahidi , A. Singhal , and H. Sotoudeh . 2023. “Radiomics; a Potential Next “Omics” in Psychiatric Disorders; an Introduction.” Psychiatry Investigation 20, no. 7: 583–592. 10.30773/pi.2022.0336. 37409371 PMC10397773

[brb371571-bib-0002] Ashok, A. H. , T. R. Marques , S. Jauhar , et al. 2017. “The Dopamine Hypothesis of Bipolar Affective Disorder: The state of the Art and Implications for Treatment.” Molecular Psychiatry 22, no. 5: 666–679. 10.1038/mp.2017.16. 28289283 PMC5401767

[brb371571-bib-0003] Babyak, M. A. 2004. “What You See May Not be What You Get: A Brief, Nontechnical Introduction to Overfitting in Regression‐Type Models.” Psychosomatic Medicine 66, no. 3: 411–421. 15184705 10.1097/01.psy.0000127692.23278.a9

[brb371571-bib-0004] Belujon, P. , and A. A. Grace . 2017. “Dopamine System Dysregulation in Major Depressive Disorders.” International Journal of Neuropsychopharmacology 20, no. 12: 1036–1046. 10.1093/ijnp/pyx056. 29106542 PMC5716179

[brb371571-bib-0005] Chapelle, O. , P. Haffner , and V. N. Vapnik . 1999. “Support Vector Machines for Histogram‐Based Image Classification.” IEEE Transactions on Neural Networks 10, no. 5: 1055–1064. 10.1109/72.788646. 18252608

[brb371571-bib-0006] Furey, T. S. , N. Cristianini , N. Duffy , D. W. Bednarski , M. Schummer , and D. Haussler . 2000. “Support Vector Machine Classification and Validation of Cancer Tissue Samples Using Microarray Expression Data.” Bioinformatics 16, no. 10: 906–914. 11120680 10.1093/bioinformatics/16.10.906

[brb371571-bib-0007] Gantz, S. C. , C. P. Ford , H. Morikawa , and J. T. Williams . 2018. “The Evolving Understanding of Dopamine Neurons in the Substantia Nigra and Ventral Tegmental Area.” Annual Review of Physiology 80: 219–241. 10.1146/annurev-physiol-021317-121615. 28938084

[brb371571-bib-0008] Gillies, R. J. , P. E. Kinahan , and H. Hricak . 2016. “Radiomics: Images Are More Than Pictures, They Are Data.” Radiology 278, no. 2: 563–577. 10.1148/radiol.2015151169. 26579733 PMC4734157

[brb371571-bib-0009] Grace, A. A. 2016. “Dysregulation of the Dopamine System in the Pathophysiology of Schizophrenia and Depression.” Nature Reviews Neuroscience 17, no. 8: 524–532. 10.1038/nrn.2016.57. 27256556 PMC5166560

[brb371571-bib-0010] Hirschfeld, R. M. , L. Lewis , and L. A. Vornik . 2003. “Perceptions and Impact of Bipolar Disorder: How Far Have We Really Come? Results of the National Depressive and Manic‐Depressive Association 2000 Survey of Individuals With Bipolar Disorder.” Journal of Clinical Psychiatry 64, no. 2: 161–174. 10.4088/JCP.v64n0209. 12633125

[brb371571-bib-0011] Huang, S. , N. Cai , P. P. Pacheco , S. Narrandes , Y. Wang , and W. Xu . 2018. “Applications of Support Vector Machine (SVM) Learning in Cancer Genomics.” Cancer Genomics & Proteomics 15, no. 1: 41–51. 29275361 10.21873/cgp.20063 PMC5822181

[brb371571-bib-0012] Huynh, P.‐H. , V. H. Nguyen , and T.‐N. Do . 2020. “Improvements in the Large *p*, Small *n* Classification Issue.” SN Computer Science 1, no. 4: 207. 10.1007/s42979-020-00210-2.

[brb371571-bib-0013] Insel, T. , B. Cuthbert , M. Garvey , et al. 2010. “Research Domain Criteria (RDoC): Toward a New Classification Framework for Research on Mental Disorders.” American Journal of Psychiatry 167, no. 7: 748–751. 10.1176/appi.ajp.2010.09091379. 20595427

[brb371571-bib-0014] Jiang, X. , B. Cao , C. Li , et al. 2024. “Identifying Misdiagnosed Bipolar Disorder Using Support Vector Machine: Feature Selection Based on fMRI of Follow‐Up Confirmed Affective Disorders.” Translational Psychiatry 14, no. 1: 9. 10.1038/s41398-023-02703-z. 38191549 PMC10774279

[brb371571-bib-0015] Kong, F. , Z. Xu , G. Yang , et al. 2024. “Microelectrode Arrays for Detection of Neural Activity in Depressed Rats: Enhanced Theta Activity in the Basolateral Amygdala.” Cyborg and Bionic Systems 5: 0125. 10.34133/cbsystems.0125. 38841725 PMC11151173

[brb371571-bib-0016] Kuai, X. , D. Shao , S. Wang , P. Y. Wu , Y. Wu , and X. Wang . 2024. “Neuromelanin‐Sensitive MRI of the Substantia Nigra Distinguishes Bipolar From Unipolar Depression.” Cerebral Cortex 34, no. 1: bhad423. 10.1093/cercor/bhad423. 37955650

[brb371571-bib-0017] Kumar, V. , Y. Gu , S. Basu , et al. 2012. “Radiomics: The Process and the Challenges.” Magnetic Resonance Imaging 30, no. 9: 1234–1248. 10.1016/j.mri.2012.06.010. 22898692 PMC3563280

[brb371571-bib-0018] Lambin, P. , R. T. H. Leijenaar , T. M. Deist , et al. 2017. “Radiomics: The Bridge Between Medical Imaging and Personalized Medicine.” Nature Reviews Clinical Oncology 14, no. 12: 749–762. 10.1038/nrclinonc.2017.141. 28975929

[brb371571-bib-0019] Lambin, P. , E. Rios‐Velazquez , R. Leijenaar , et al. 2012. “Radiomics: Extracting More Information From Medical Images Using Advanced Feature Analysis.” European Journal of Cancer 48, no. 4: 441–446. 10.1016/j.ejca.2011.11.036. 22257792 PMC4533986

[brb371571-bib-0020] Le, G. H. , S. Wong , H. Au , et al. 2025. “Association Between Rumination, Suicidal Ideation and Suicide Attempts in Persons With Depressive and Other Mood Disorders and Healthy Controls: A Systematic Review and Meta‐Analysis.” Journal of Affective Disorders 368: 513–527. 10.1016/j.jad.2024.09.118. 39303880

[brb371571-bib-0021] Le‐Niculescu, H. , K. Roseberry , S. S. Gill , et al. 2021. “Precision Medicine for Mood Disorders: Objective Assessment, Risk Prediction, Pharmacogenomics, and Repurposed Drugs.” Molecular Psychiatry 26, no. 7: 2776–2804. 10.1038/s41380-021-01061-w. 33828235 PMC8505261

[brb371571-bib-0022] Li, M. , X. Li , Y. Guo , et al. 2020. “Development and Assessment of an Individualized Nomogram to Predict Colorectal Cancer Liver Metastases.” Quantitative Imaging in Medicine and Surgery 10, no. 2: 397–414. 10.21037/qims.2019.12.16. 32190566 PMC7063284

[brb371571-bib-0023] Northoff, G. , D. Hirjak , R. C. Wolf , P. Magioncalda , and M. Martino . 2021. “All Roads Lead to the Motor Cortex: Psychomotor Mechanisms and Their Biochemical Modulation in Psychiatric Disorders.” Molecular Psychiatry 26, no. 1: 92–102. 10.1038/s41380-020-0814-5. 32555423

[brb371571-bib-0024] Phillips, M. L. , and D. J. Kupfer . 2013. “Bipolar Disorder Diagnosis: Challenges and Future Directions.” Lancet 381, no. 9878: 1663–1671. 10.1016/S0140-6736(13)60989-7. 23663952 PMC5858935

[brb371571-bib-0025] Sekhon, S. , and V. Gupta . 2025. “Mood Disorder.” In StatPearls. StatPearls Publishing. 32644337

[brb371571-bib-0026] Shinde, S. , S. Prasad , Y. Saboo , et al. 2019. “Predictive Markers for Parkinson's Disease Using Deep Neural Nets on Neuromelanin Sensitive MRI.” NeuroImage: Clinical 22: 101748. 10.1016/j.nicl.2019.101748. 30870733 PMC6417260

[brb371571-bib-0027] Siegel‐Ramsay, J. E. , M. A. Bertocci , B. Wu , M. L. Phillips , S. M. Strakowski , and J. R. C. Almeida . 2022. “Distinguishing Between Depression in Bipolar Disorder and Unipolar Depression Using Magnetic Resonance Imaging: A Systematic Review.” Bipolar Disorders 24, no. 5: 474–498. 10.1111/bdi.13176. 35060259

[brb371571-bib-0028] Statnikov, A. , L. Wang , and C. F. Aliferis . 2008. “A Comprehensive Comparison of Random Forests and Support Vector Machines for Microarray‐Based Cancer Classification.” BMC Bioinformatics 9: 319. 10.1186/1471-2105-9-319. 18647401 PMC2492881

[brb371571-bib-0029] Sun, K. , G. Chen , C. Liu , et al. 2025. “A Novel MSN‐II Feature Extracted From T1‐Weighted MRI for Discriminating Between BD Patients and MDD Patients.” Journal of Affective Disorders 371: 36–44. 10.1016/j.jad.2024.11.047. 39557301

[brb371571-bib-0030] Trujillo, P. , M. A. Aumann , and D. O. Claassen . 2024. “Neuromelanin‐Sensitive MRI as a Promising Biomarker of Catecholamine Function.” Brain 147, no. 2: 337–351. 10.1093/brain/awad300. 37669320 PMC10834262

[brb371571-bib-0031] Xu, L. , P. Yang , W. Liang , et al. 2019. “A Radiomics Approach Based on Support Vector Machine Using MR Images for Preoperative Lymph Node Status Evaluation in Intrahepatic Cholangiocarcinoma.” Theranostics 9, no. 18: 5374–5385. 10.7150/thno.34149. 31410221 PMC6691572

[brb371571-bib-0032] Zhong, R. , X. Wu , J. Chen , and Y. Fang . 2025. “Using Digital Phenotyping to Discriminate Unipolar Depression and Bipolar Disorder: Systematic Review.” Journal of Medical Internet Research 27: e72229. 10.2196/72229. 40408762 PMC12144479

