# Supplementary material for: Radiomic Signature of the Substantia Nigra on Neuromelanin‐Sensitive MRI Distinguishes Bipolar II Disorder From Unipolar Depression
Source: Brain Behav. 2026 Jul 8;16(7):e71571. doi: 10.1002/brb3.71571 (PMC13344892; doi:10.1002/brb3.71571)

*Neuroimaging Data Acquisition and Data Preprocessing*

MR images of the brain were acquired for all participants on a 3 T MR Scanner (MAGNETOM Verio, Siemens Healthcare, Erlangen, Germany) with a 32-channel head coil using a 2D GRE sequence with magnetization transfer contrast (MTC). The imaging parameters were chosen as follows: repetition time (TR) = 273 ms; echo time (TE) = 3.87 ms; f lip angle = 40◦; partial brain coverage with field of view (FOV) = 220 × 165; matrix = 242 × 512; number of slices = 9; slice thickness = 3 mm; slice gap = 0 mm; magnetization transfer frequency offset = 1,200 Hz; number of excitations (NEX) = 10; acquisition time = 11:02 min. The imaging region was centered on the pons and positioned parallel to the anterior commissure-posterior commissure (AC-PC) line.

The NM-MRI images were first converted into the Neuroimaging Informatics Technology Initiative (NIFTI) format using MRIcroGL software (version 1.2.20210317) before segmentation (https://www.nitrc.org/projects/mricrogl). Next, all regions of interest (ROIs) and volumes of interest were manually drawn on individual NM-MRI, by two experienced radiologists with >10 (rater 1, X. Kuai) and five (rater 2, X. Wang) years of experience in neuroimaging, respectively. Both raters were fully blinded to all clinical diagnoses and group assignments. ROI sets of the SN were segmented in three consecutive axial slices that were internal to the cerebral peduncle with the best contrast around this region from each participant. To mitigate intensity inhomogeneity and standardize intensity scales, all MRI scans underwent N4 bias field correction and image intensity normalization. Prior to radiomic feature extraction, all images were resampled to an isotropic voxel size of 1 × 1 × 1 mm³ using B‑spline interpolation (default setting of the Radiomics Intelligent Analysis Toolkit, RIAS). Following resampling, the following 15 image types were generated for each subject: (1) the original image; (2) voxel-wise transformations including exponential, logarithmic, square, and square root; (3) Laplacian of Gaussian (LoG) filters with sigma parameters of 1.0 mm and 3.0 mm (equivalent to 1 and 3 voxels, respectively, after resampling); and (4) eight wavelet-decomposed subbands with single-level decomposition. All image filtering and transformation procedures were performed using RIAS (version 1.1.1).

*Fully Nested Leave-One-Out Cross-Validation (LOOCV)*

*Methodological Overview*

To validate the generalizability of our radiomic signature and eliminate potential data leakage, we implemented a strict fully nested leave-one-out cross-validation (LOOCV) framework using a linear support vector machine (SVM) classifier, which adheres to the following core principles: The held-out test sample was completely excluded from all preprocessing, feature selection, and model training steps in each outer LOOCV fold. All feature selection procedures (ANOVA filtering, correlation-based redundancy removal, LASSO regularization) were conducted exclusively on the training subset within each fold. The final classification model was trained on the LASSO-selected features from the training set, and only then applied to the independent test sample.

*Overall Model Performance*

Overall accuracy in fully nested LOOCV was 0.611 (95% CI: 0.520–0.697), and multiclass AUC was 0.734.

The complete confusion matrix for the three-class classification is presented below:


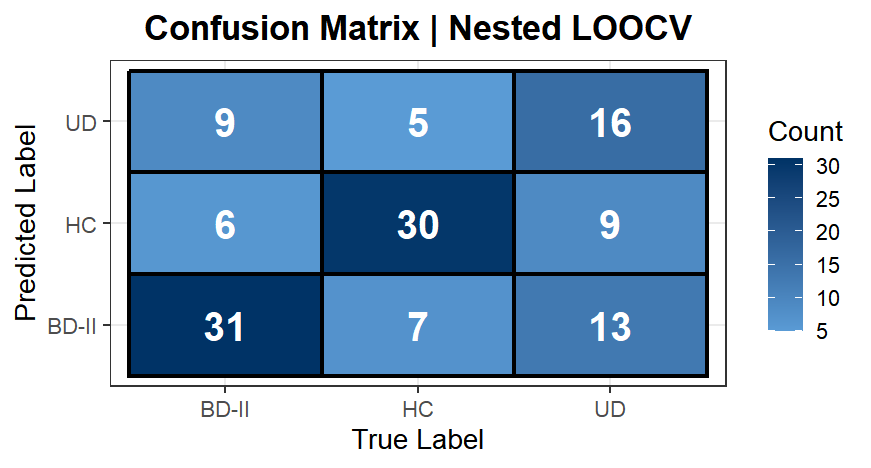

Supplement: Supplementary file 1 — Supplementary Materials: brb371571‐sup‐0001‐SuppMat.docx [file BRB3-16-e71571-s001.docx]
